# Supplementary material for: A Likelihood Approach for Real-Time Calibration of Stochastic Compartmental Epidemic Models
Source: PLoS Comput Biol. 2017 Jan 17;13(1):e1005257. doi: 10.1371/journal.pcbi.1005257 (PMC5240920; doi:10.1371/journal.pcbi.1005257)
Supplement: S1 File — (TAR.GZ) [file pcbi.1005257.s014.tar.gz › HSPH_Online-SI-Revision/Additions/IPoi10/n100-extreme-SEITR_noisy/n100-extreme-SEITR_noisy_BENCH_table0.pdf]

1	R0	{1.38654, 9.85565}	{2.93978, 19.9817}	{{2.93978, 19.9817}, {2.93978, 19.9817}}	{112.023, 102.744}	inf	5261.	25118.4	{19365., 29171.}	37.6777	0.138291	newly	12047.4	8506.52	{6459., 10184.}	29.391	0.158135	Reff	0.312221	0.885007	{0.456754, 1.60856}	183.455	ar	92379.	93630.7	{93491., 93770.}	1.355
2	R0	{2.36629, 12.2029}	{2.91946, 11.8546}	{{2.91946, 11.8546}, {2.91946, 11.8546}}	{23.3771, 2.85424}	inf	4672.	24608.6	{19823., 28929.}	29.7057	0.185756	newly	17453.	14083.9	{10634., 16616.}	19.3038	0.115132	Reff	0.202507	1.09269	{0.537706, 1.92857}	439.581	ar	99022.	93463.2	{93300., 93646.}	5.61371
3	R0	{2.32253, 12.836}	{2.98388, 9.60029}	{{2.98388, 9.60029}, {2.98388, 9.60029}}	{28.4754, 25.2081}	inf	6387.	19784.3	{12892., 27774.}	45.4948	0.278658	newly	18266.	11825.8	{7333., 17428.}	36.2437	0.213098	Reff	0.29092	0.453185	{0.297582, 0.676147}	56.0921	ar	98997.	93958.2	{93819., 94112.}	5.08983
4	R0	{1.79075, 11.2058}	{2.82352, 10.8578}	{{2.80387, 10.6536}, {2.91946, 11.8546}}	{57.6725, 5.07435}	inf	6785.	19032.9	{11952., 26119.}	31.9996	0.182392	newly	16022.	10587.8	{6360., 15196.}	34.7115	0.203348	Reff	0.341174	0.542089	{0.333941, 0.808953}	59.9656	ar	96920.	92661.4	{92365., 93440.}	4.39395
5	R0	{2.26952, 9.62371}	{2.98388, 9.60029}	{{2.98388, 9.60029}, {2.98388, 9.60029}}	{31.4763, 0.243357}	inf	2810.	24283.8	{17563., 28974.}	17.5954	0.108609	newly	16920.	16253.	{12024., 20966.}	16.1459	0.0844591	Reff	0.133902	0.913533	{0.444389, 1.94188}	582.242	ar	98881.	93931.6	{93787., 94081.}	5.00543
6	R0	{1.57191, 6.89004}	{2.9194, 10.1535}	{{2.9194, 10.1535}, {2.9194, 10.1535}}	{85.7231, 47.3649}	inf	4582.	23699.7	{16723., 28718.}	24.3575	0.107312	newly	17405.	14821.5	{10084., 19107.}	18.8406	0.0989113	Reff	0.240125	0.839366	{0.431079, 1.52013}	249.554	ar	95046.	93450.7	{93321., 93554.}	1.67844
7	R0	{1.99226, 17.2339}	{2.97157, 19.3547}	{{2.97157, 19.3547}, {2.97157, 19.3547}}	{49.1557, 12.306}	inf	5638.	23267.3	{16544., 29760.}	22.5034	0.129919	newly	11332.	7951.34	{5306., 10496.}	29.8328	0.172071	Reff	0.270071	0.759278	{0.375042, 1.19781}	181.14	ar	97992.	93848.1	{93678., 93974.}	4.2288
8	R0	{2.63369, 15.1879}	{2.83858, 10.6973}	{{2.83858, 10.6973}, {2.83858, 10.6973}}	{7.77958, 29.567}	inf	3741.	24046.5	{19832., 27584.}	34.6634	0.188658	newly	14766.	14496.2	{11308., 17697.}	13.7107	0.0620888	Reff	0.159786	0.831542	{0.490847, 1.54422}	420.41	ar	99451.	92810.5	{92617., 93012.}	6.67715
9	R0	{2.53271, 9.90552}	{2.98388, 9.60029}	{{2.98388, 9.60029}, {2.98388, 9.60029}}	{17.8137, 3.08141}	inf	2374.	25153.3	{19812., 29470.}	19.7944	0.109569	newly	17411.	16405.1	{11549., 21127.}	17.4068	0.083838	Reff	0.11382	0.821339	{0.430246, 1.41179}	621.613	ar	99355.	93929.9	{93767., 94058.}	5.46027
10	R0	{2.48791, 18.9003}	{2.7943, 13.5962}	{{2.7943, 13.5962}, {2.7943, 13.5962}}	{12.3152, 28.0636}	inf	6612.	19924.3	{13899., 26892.}	48.2096	0.298226	newly	13614.	9248.81	{6160., 13315.}	32.2006	0.186261	Reff	0.296509	0.589352	{0.359319, 0.954952}	98.7636	ar	99291.	92460.2	{92306., 92648.}	6.87961
11	R0	{2.84414, 18.4765}	{2.99911, 15.6207}	{{2.99911, 15.6207}, {2.99911, 15.6207}}	{5.44875, 15.4564}	inf	5335.	24620.6	{18544., 29645.}	41.304	0.238681	newly	14675.	10460.2	{7497., 13206.}	28.7211	0.156119	Reff	0.221814	0.812364	{0.399272, 1.57417}	266.236	ar	99654.	94029.5	{93867., 94159.}	5.64401
12	R0	{2.29369, 14.622}	{2.99939, 13.7076}	{{2.99939, 13.7076}, {2.99939, 13.7076}}	{30.767, 6.25359}	inf	4308.	24649.5	{18279., 29380.}	25.855	0.137405	newly	14159.	11658.5	{8393., 14901.}	19.3106	0.101085	Reff	0.188656	0.76226	{0.39238, 1.51388}	304.047	ar	98982.	94029.8	{93868., 94163.}	5.00312
13	R0	{2.73413, 16.7529}	{2.9884, 12.8326}	{{2.9884, 12.8326}, {2.9884, 12.8326}}	{9.29985, 23.4007}	inf	4068.	23398.7	{16970., 29337.}	40.0909	0.232669	newly	14704.	11502.6	{7982., 15432.}	24.0081	0.130293	Reff	0.173699	0.647388	{0.365003, 1.21685}	272.706	ar	99543.	93966.7	{93788., 94105.}	5.60192
14	R0	{1.79554, 16.5943}	{2.86095, 19.9929}	{{2.86095, 19.9929}, {2.86095, 19.9929}}	{59.3365, 20.4805}	inf	7555.	22342.5	{14948., 28154.}	20.9465	0.115286	newly	11642.	7399.93	{4633., 9685.}	36.4377	0.212953	Reff	0.390135	0.700067	{0.36048, 1.19493}	81.969	ar	96821.	93020.	{92875., 93190.}	3.92575
15	R0	{1.91429, 13.7771}	{2.97157, 19.3547}	{{2.97157, 19.3547}, {2.97157, 19.3547}}	{55.2309, 40.4846}	inf	5519.	23392.7	{18485., 28827.}	18.9188	0.102654	newly	13230.	8035.97	{5974., 10316.}	39.2595	0.228931	Reff	0.264038	0.745478	{0.399765, 1.42716}	182.337	ar	97541.	93854.7	{93736., 93959.}	3.7792
16	R0	{1.42094, 10.5871}	{2.86118, 15.5722}	{{2.86118, 15.5722}, {2.86118, 15.5722}}	{101.358, 47.0865}	inf	7009.	18942.	{12280., 26129.}	22.4393	0.109572	newly	12953.	7663.69	{4753., 10859.}	40.8346	0.250509	Reff	0.417131	0.508146	{0.315388, 0.705481}	37.0796	ar	93044.	92995.3	{92864., 93140.}	0.121448
17	R0	{1.45515, 6.16466}	{2.98388, 9.60029}	{{2.98388, 9.60029}, {2.98388, 9.60029}}	{105.057, 55.7311}	inf	6217.	24602.8	{18121., 29542.}	28.3092	0.119105	newly	20574.	16384.5	{11083., 20944.}	21.1764	0.114127	Reff	0.356002	0.895586	{0.431887, 1.6522}	151.567	ar	93254.	93909.8	{93749., 94083.}	0.703287
18	R0	{2.85669, 13.2416}	{2.9194, 10.1535}	{{2.9194, 10.1535}, {2.9194, 10.1535}}	{2.1952, 23.3212}	inf	3137.	24207.1	{19182., 28503.}	36.8603	0.207324	newly	17189.	14924.	{11055., 19407.}	17.5168	0.0875675	Reff	0.132179	0.765872	{0.437881, 1.25146}	479.42	ar	99644.	93437.9	{93300., 93602.}	6.22823
19	R0	{2.56417, 17.9558}	{2.86118, 15.5722}	{{2.86118, 15.5722}, {2.86118, 15.5722}}	{11.5831, 13.2748}	inf	6398.	24725.3	{21207., 28200.}	37.5198	0.224858	newly	14542.	10669.3	{8921., 12458.}	26.6315	0.1497	Reff	0.282084	0.957006	{0.556185, 1.63179}	239.262	ar	99389.	93027.8	{92867., 93168.}	6.40027
20	R0	{2.27517, 11.1864}	{2.9194, 10.1535}	{{2.9194, 10.1535}, {2.9194, 10.1535}}	{28.3157, 9.23353}	inf	3799.	24028.9	{18558., 28954.}	24.8204	0.157581	newly	16864.	14775.	{11396., 18944.}	17.7474	0.109086	Reff	0.172117	0.795551	{0.437005, 1.23365}	362.216	ar	98894.	93467.8	{93294., 93639.}	5.48686
21	R0	{2.35735, 10.4735}	{2.98388, 9.60029}	{{2.98388, 9.60029}, {2.98388, 9.60029}}	{26.5777, 8.33733}	inf	3960.	24811.	{19027., 29602.}	25.6734	0.169179	newly	18543.	16382.5	{11733., 20707.}	18.1949	0.112714	Reff	0.172039	0.891029	{0.445792, 1.66283}	417.921	ar	99036.	93946.9	{93803., 94058.}	5.13867
22	R0	{1.79551, 11.9179}	{2.99939, 13.7076}	{{2.99939, 13.7076}, {2.99939, 13.7076}}	{67.0495, 15.0169}	inf	5574.	15672.8	{8820., 25644.}	42.6273	0.26912	newly	14003.	6945.41	{3703., 11540.}	50.661	0.343639	Reff	0.270045	0.396891	{0.249549, 0.615715}	49.8797	ar	97033.	94033.7	{93879., 94203.}	3.09105
23	R0	{1.23545, 6.31687}	{2.9884, 12.8326}	{{2.9884, 12.8326}, {2.9884, 12.8326}}	{141.888, 103.148}	inf	4400.	25898.7	{21482., 29675.}	90.1264	0.3046	newly	13260.	13331.7	{10127., 15951.}	15.3792	0.0939041	Reff	0.306775	0.927504	{0.480146, 1.57088}	202.34	ar	88739.	93967.6	{93801., 94128.}	5.89213
24	R0	{2.24089, 10.1789}	{2.83858, 10.6973}	{{2.83858, 10.6973}, {2.83858, 10.6973}}	{26.672, 5.09289}	inf	5491.	24474.7	{18963., 27941.}	27.551	0.15535	newly	20012.	15964.4	{12227., 18004.}	20.2257	0.10438	Reff	0.242442	1.19805	{0.671012, 1.90835}	394.16	ar	98868.	92820.4	{92688., 92992.}	6.11681
25	R0	{1.57298, 10.2607}	{2.99911, 15.6207}	{{2.99911, 15.6207}, {2.99911, 15.6207}}	{90.6642, 52.2382}	inf	7361.	25572.3	{17617., 29771.}	21.147	0.101383	newly	15457.	11212.6	{8321., 13243.}	27.4593	0.152301	Reff	0.403627	1.06886	{0.534741, 2.0492}	165.036	ar	94957.	94055.4	{93884., 94179.}	0.949476
26	R0	{1.2154, 8.2576}	{2.8939, 16.8013}	{{2.8939, 16.8013}, {2.8939, 16.8013}}	{138.103, 103.465}	inf	5404.	24421.2	{19559., 28558.}	64.9288	0.215698	newly	11730.	10125.	{7378., 11880.}	14.2527	0.0743987	Reff	0.35796	1.08991	{0.46369, 1.90618}	204.478	ar	88421.	93280.4	{93096., 93424.}	5.49578
27	R0	{2.22841, 18.7983}	{2.8939, 16.8013}	{{2.8939, 16.8013}, {2.8939, 16.8013}}	{29.8639, 10.6233}	inf	5503.	19304.2	{13009., 27018.}	43.318	0.262216	newly	11908.	7350.06	{4743., 10989.}	38.3031	0.230535	Reff	0.242317	0.541153	{0.325535, 0.865652}	123.324	ar	98756.	93265.8	{93147., 93406.}	5.55934
28	R0	{1.27778, 5.36767}	{2.98388, 9.60029}	{{2.98388, 9.60029}, {2.98388, 9.60029}}	{133.521, 78.854}	inf	5294.	20997.1	{13818., 28521.}	35.4533	0.130696	newly	18063.	12956.9	{8147., 18532.}	30.7588	0.174981	Reff	0.340107	0.574448	{0.315068, 0.971939}	72.1612	ar	89843.	93959.4	{93815., 94113.}	4.58175
29	R0	{2.38042, 15.8901}	{2.9884, 12.8326}	{{2.9884, 12.8326}, {2.9884, 12.8326}}	{25.5409, 19.2415}	inf	6676.	17119.	{10800., 26307.}	53.9948	0.359184	newly	15388.	8048.99	{4818., 12988.}	47.7684	0.309465	Reff	0.307407	0.430192	{0.270151, 0.645584}	45.612	ar	99112.	93970.4	{93840., 94118.}	5.18769
30	R0	{2.53188, 12.7607}	{2.9194, 10.1535}	{{2.9194, 10.1535}, {2.9194, 10.1535}}	{15.3056, 20.4315}	inf	7079.	25203.3	{18252., 29053.}	35.742	0.216239	newly	19690.	17333.	{14221., 19568.}	12.0638	0.068613	Reff	0.319143	1.22707	{0.687198, 2.01658}	284.488	ar	99317.	93468.2	{93287., 93638.}	5.88905
31	R0	{1.24482, 10.5471}	{2.93978, 19.9817}	{{2.93978, 19.9817}, {2.93978, 19.9817}}	{136.161, 89.4521}	inf	5495.	23838.	{18799., 28650.}	54.1074	0.188834	newly	9931.	7983.27	{5971., 9901.}	20.0743	0.109698	Reff	0.364384	0.797816	{0.40716, 1.43088}	119.425	ar	89097.	93608.9	{93426., 93773.}	5.06404
32	R0	{2.30446, 17.9607}	{2.95209, 14.037}	{{2.95209, 14.037}, {2.95209, 14.037}}	{28.1033, 21.846}	inf	6256.	23645.	{17217., 29309.}	33.8712	0.194262	newly	13001.	10878.3	{7221., 14341.}	20.9043	0.111736	Reff	0.270705	0.734729	{0.368598, 1.12767}	171.413	ar	99028.	93713.	{93562., 93875.}	5.36714
33	R0	{2.31579, 11.8236}	{2.9751, 8.95585}	{{2.9751, 8.95585}, {2.9751, 8.95585}}	{28.4702, 24.2545}	inf	3005.	9702.33	{5374., 15651.}	68.0456	0.539733	newly	15166.	5849.05	{3154., 9468.}	63.246	0.471339	Reff	0.14124	0.278652	{0.220663, 0.344487}	97.2894	ar	99012.	93873.6	{93724., 94037.}	5.18963
34	R0	{1.6678, 10.6744}	{2.99939, 13.7076}	{{2.99939, 13.7076}, {2.99939, 13.7076}}	{79.8411, 28.4156}	inf	4965.	22427.9	{15920., 28907.}	16.5401	0.0774143	newly	13645.	10394.8	{7358., 14249.}	25.8759	0.139949	Reff	0.259643	0.640989	{0.355728, 1.26001}	146.873	ar	95809.	94057.2	{93906., 94206.}	1.82845
35	R0	{1.65476, 7.07559}	{2.98388, 9.60029}	{{2.98388, 9.60029}, {2.98388, 9.60029}}	{80.321, 35.6818}	inf	5229.	25245.3	{20287., 29800.}	14.7955	0.0620656	newly	19217.	16434.7	{12548., 20744.}	18.5171	0.0941809	Reff	0.270404	0.77486	{0.442718, 1.39869}	186.556	ar	95746.	93941.2	{93804., 94085.}	1.88498
36	R0	{2.71807, 18.5959}	{2.7943, 13.5962}	{{2.7943, 13.5962}, {2.7943, 13.5962}}	{2.80456, 26.886}	inf	7257.	23576.5	{17914., 27229.}	43.6831	0.265288	newly	15024.	12112.9	{10014., 13936.}	19.3765	0.102361	Reff	0.322961	1.15951	{0.615696, 1.91038}	259.024	ar	99540.	92446.8	{92256., 92624.}	7.12598
37	R0	{2.6202, 12.7552}	{2.99939, 13.7076}	{{2.99939, 13.7076}, {2.99939, 13.7076}}	{14.4718, 7.46676}	inf	5145.	26204.8	{17827., 29986.}	33.0331	0.188338	newly	18951.	13265.	{11100., 15199.}	30.0035	0.161885	Reff	0.212734	1.15535	{0.612355, 2.10773}	443.098	ar	99426.	94041.2	{93918., 94170.}	5.41591
38	R0	{1.47672, 9.81651}	{2.86118, 15.5722}	{{2.86118, 15.5722}, {2.86118, 15.5722}}	{93.7524, 58.6328}	inf	5442.	22478.5	{16581., 28022.}	20.0229	0.0880543	newly	13535.	9371.33	{6368., 12386.}	30.7622	0.17374	Reff	0.299449	0.719594	{0.399163, 1.16281}	140.306	ar	93815.	93016.7	{92866., 93192.}	0.850885
39	R0	{1.90004, 12.3313}	{2.96307, 14.3219}	{{2.96307, 14.3219}, {2.96307, 14.3219}}	{55.9478, 16.1427}	inf	6237.	23702.2	{17510., 29021.}	18.0649	0.0929873	newly	15042.	10580.1	{7268., 13281.}	29.6628	0.16311	Reff	0.303056	0.662355	{0.375865, 1.11408}	118.558	ar	97413.	93802.9	{93630., 93981.}	3.70596
40	R0	{2.64218, 18.5237}	{2.9884, 12.8326}	{{2.9884, 12.8326}, {2.9884, 12.8326}}	{13.1036, 30.7233}	inf	4328.	16499.2	{9686., 27212.}	56.4264	0.388139	newly	13194.	7770.89	{4361., 13773.}	43.5855	0.274695	Reff	0.17626	0.415938	{0.261156, 0.727526}	135.98	ar	99474.	93985.3	{93829., 94146.}	5.51772
41	R0	{1.6945, 7.17996}	{2.9194, 10.1535}	{{2.9194, 10.1535}, {2.9194, 10.1535}}	{72.2868, 41.4144}	inf	7462.	24253.8	{13712., 28980.}	16.0045	0.0925855	newly	23349.	17327.3	{13735., 19662.}	25.7898	0.137994	Reff	0.379415	1.371	{0.732331, 2.26674}	261.346	ar	96332.	93461.6	{93341., 93618.}	2.97967
42	R0	{1.84559, 18.0074}	{2.97157, 19.3547}	{{2.97157, 19.3547}, {2.97157, 19.3547}}	{61.0092, 7.48192}	inf	6919.	24609.9	{18979., 29573.}	15.4942	0.0808237	newly	10541.	8333.24	{6179., 10352.}	20.9445	0.110859	Reff	0.338389	0.691616	{0.401429, 0.928764}	104.385	ar	97358.	93877.8	{93735., 94062.}	3.57465
43	R0	{2.24965, 17.0361}	{2.7943, 13.5962}	{{2.7943, 13.5962}, {2.7943, 13.5962}}	{24.2104, 20.1918}	inf	7490.	20170.3	{14625., 26837.}	42.8799	0.259177	newly	14237.	9558.87	{6615., 13355.}	32.859	0.189723	Reff	0.339337	0.72244	{0.388715, 1.44921}	112.897	ar	98770.	92428.3	{92248., 92570.}	6.42066
44	R0	{2.54324, 15.7967}	{2.95209, 14.037}	{{2.95209, 14.037}, {2.95209, 14.037}}	{16.076, 11.1397}	inf	3895.	26087.4	{20369., 29413.}	28.0327	0.152023	newly	14170.	12653.6	{10421., 14455.}	11.5754	0.0590562	Reff	0.167193	1.02089	{0.560602, 1.87942}	510.607	ar	99354.	93710.4	{93572., 93830.}	5.68026
45	R0	{2.54943, 14.8032}	{2.83858, 10.6973}	{{2.83858, 10.6973}, {2.83858, 10.6973}}	{11.3418, 27.7366}	inf	4134.	22860.9	{17622., 27710.}	36.9841	0.207551	newly	14996.	13618.2	{9606., 17667.}	18.5848	0.0913139	Reff	0.179352	0.772873	{0.419769, 1.4636}	330.924	ar	99380.	92865.2	{92707., 93042.}	6.55543
46	R0	{2.37755, 12.6461}	{2.98388, 9.60029}	{{2.98388, 9.60029}, {2.98388, 9.60029}}	{25.5023, 24.085}	inf	5896.	14633.9	{8075., 22141.}	59.6385	0.428658	newly	17991.	8762.87	{4497., 15881.}	52.6854	0.363802	Reff	0.261673	0.400141	{0.245603, 0.649262}	55.4717	ar	99096.	93917.1	{93781., 94066.}	5.22618
47	R0	{1.69279, 11.6636}	{2.95376, 15.278}	{{2.95376, 15.278}, {2.95376, 15.278}}	{74.4906, 30.9887}	inf	6849.	24287.5	{18836., 29088.}	12.7022	0.0599493	newly	14547.	10468.9	{7839., 13193.}	28.0338	0.151426	Reff	0.3499	0.772255	{0.412699, 1.52698}	120.968	ar	96214.	93716.2	{93599., 93849.}	2.59612
48	R0	{1.82402, 10.0038}	{2.9884, 12.8326}	{{2.9884, 12.8326}, {2.9884, 12.8326}}	{63.8359, 28.2773}	inf	6918.	25393.5	{19276., 29652.}	12.7846	0.0794691	newly	17877.	12841.	{8836., 15964.}	28.1704	0.164026	Reff	0.343281	0.827606	{0.407648, 1.27288}	141.087	ar	97076.	93949.4	{93820., 94067.}	3.22073
49	R0	{1.6424, 11.1667}	{2.99939, 13.7076}	{{2.99939, 13.7076}, {2.99939, 13.7076}}	{82.6224, 22.7543}	inf	4677.	16579.5	{10116., 25867.}	32.7448	0.181895	newly	12499.	7345.47	{4253., 11762.}	43.841	0.27118	Reff	0.249793	0.403258	{0.266886, 0.597209}	61.6059	ar	95546.	94037.8	{93876., 94179.}	1.57849
50	R0	{2.05638, 14.3048}	{2.9884, 12.8326}	{{2.9884, 12.8326}, {2.9884, 12.8326}}	{45.3233, 10.2917}	inf	6276.	22471.	{15675., 29340.}	28.8712	0.160035	newly	14245.	11053.5	{7240., 15392.}	25.5765	0.139853	Reff	0.290299	0.638049	{0.334253, 1.0235}	119.799	ar	98194.	93966.4	{93822., 94129.}	4.30538
